# PM2.5 Induces Early Epithelial Mesenchymal Transition in Human Proximal Tubular Epithelial Cells through Activation of IL-6/STAT3 Pathway

**DOI:** 10.3390/ijms222312734

**Published:** 2021-11-25

**Authors:** Chien-Hung Lin, Chuan Wan, Wen-Sheng Liu, Hsin-Hui Wang

**Affiliations:** 1 Division of Pediatric Immunology and Nephrology, Department of Pediatrics, Taipei Veterans General Hospital, Taipei 112201, Taiwan; chlin5@vghtpe.gov.tw; 2 Institute of Clinical Medicine, National Yang Ming Chiao Tung University, Taipei 112304, Taiwan; DAC78@tpech.gov.tw; 3 College of Science and Engineering, Fu Jen Catholic University, New Taipei 242062, Taiwan; robertliu2001@yahoo.com; 4 Department of Pediatrics, Taipei City Hospital, Zhongxing Branch, Taipei 10341, Taiwan; 5 Division of Nephrology, Department of Medicine, Taipei City Hospital, ZhongXing Branch, Taipei 10341, Taiwan; 6 Faculty of Medicine, School of Medicine, National Yang Ming Chiao Tung University, Hsinchu 300093, Taiwan; 7 Institute of Food Safety and Health Risk Assessment, National Yang Ming Chiao Tung University, Hsinchu 300093, Taiwan; 8 Institute of Emergency and Critical Care Medicine, National Yang Ming Chiao Tung University, Taipei 112304, Taiwan

**Keywords:** PM2.5, interleukin-6, epithelial–mesenchymal transition, STAT3

## Abstract

Particulate matter exposure has been known as a potential risk for the global burden of disease, such as respiratory and cardiovascular diseases. Accumulating evidence suggests that PM2.5 (particulate matter with a diameter less than 2.5 μm) is associated with increased risk of kidney disease, but the mechanisms underlying the renal injury caused by PM2.5 remain to be elucidated. This study investigated the effects of PM2.5 on human proximal tubular epithelial (HK-2) cells by monolayer and 3D spheroid cultures and explored the potential mechanisms. The typical morphology of HK-2 cells showed epithelial–mesenchymal transition (EMT), resulting in reduced adhesion and enhanced migration after PM2.5 exposure, and was accompanied by decreased E-cadherin expression and increased vimentin and α-SMA expressions. Exposure to PM2.5 in the HK-2 cells could lead to an increase in interleukin-6 (IL-6) levels and cause the activation of signal transducer and activator of transcription 3 (STAT3), which is involved in EMT features of HK-2 cells. Furthermore, blocking IL-6/STAT3 signaling by an IL-6 neutralizing antibody or STAT3 inhibitor was sufficient to reverse PM2.5-induced EMT characteristics of the HK-2 cells. Our study suggests that PM2.5 could induce early renal tubule cell injury, contributing to EMT change, and the induction of IL-6/STAT3 pathway may play an important role in this process.

## 1. Introduction

Air pollutants are major environmental substances that adversely affect human health globally [1,2]. Fine particulate matter (PM2.5) is a component of air pollutants with a diameter of less than 2.5 micrometers that can permeate the lung alveoli, enter the blood circulation, and induce a systemic inflammatory response [3,4]. Long-term exposure to PM2.5 has significant negative health impacts, and the risks of morbidity and mortality are found to be related to short-term exposure to PM2.5 as well. [5,6]. PM2.5 may contribute to various human illnesses such as respiratory disease, cardiovascular disease, brain disorder progression, diabetes mellitus, and cancer [7,8,9,10,11]. Recent epidemiological studies suggest an association between chronic kidney disease (CKD), end-stage renal disease (ESRD), and exposure to air pollution [12,13,14]. However, the effects of PM2.5 on the kidney and the underlying molecular mechanism associated with increased risk of developing kidney disease have not been fully established.

Epithelial–mesenchymal transition (EMT) is a cellular process during which epithelial cells gradually acquire mesenchymal characteristics and lost their epithelial phenotype. The renal tubule is a primary site of nephrotoxicity and proximal tubular epithelial injury alone could induce interstitial fibrosis and cause CKD [15,16]. The occurrence of EMT in the renal tubule epithelial cells during early renal injury is associated with tubulointerstitial fibrosis, and the reversal of EMT may slow the progression of CKD [17,18,19]. The production of the cytokine interleukin (IL)-6 is a cell response triggered by various toxic environmental compounds, including PM2.5. Several studies have shown that CKD is associated with increased IL-6 expression and activation of STAT3 [20,21]. Additionally, it has been reported that IL-6/STAT3 signaling pathway is involved in the EMT of injured cells and organ fibrosis [22,23]. 

The adverse impact of PM2.5 is becoming a rising health threat worldwide. However, the effects and mechanism underlying the association between PM2.5 and the development of renal injury are not fully understood. This study aimed to investigate the effects of PM2.5 on human HK-2 cells and examine the role of IL-6/STAT3 pathway in PM2.5-induced EMT.

## 2. Results

### 2.1. Effects of PM2.5 on Cell Viability and Morphological Changes of HK-2 Cells

**Figure 1:**
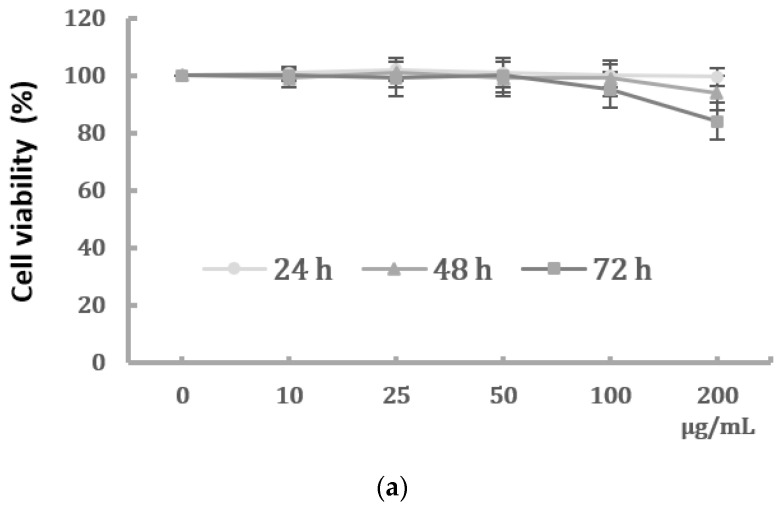

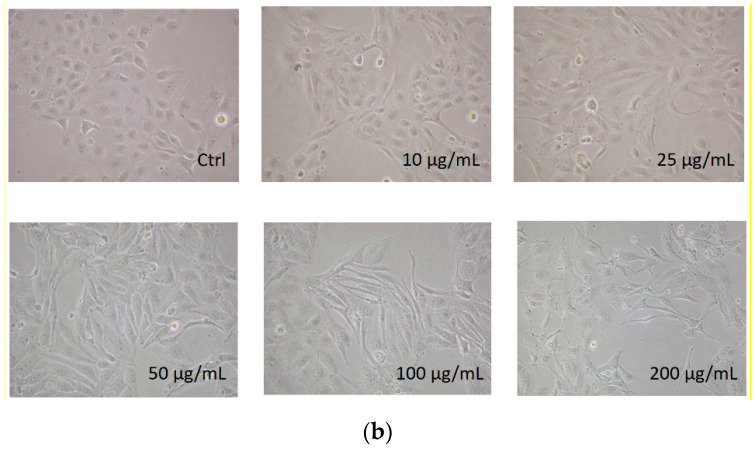
Effects of PM2.5 on cell viability and phenotype of HK-2 cells. (**a**) Cell viability of HK-2 cells following different concentrations of PM2.5 exposure for 24, 48, and 72 h using the MTT assay. Data are presented as the mean ± SD of at least three independent experiments. (**b**) Phenotype changes of HK-2 cells following different concentrations of PM2.5 exposure (original magnification ×100).

We first determined the dose-dependent effects of PM2.5 on the cell viability of HK-2 cells. HK-2 cells were treated with various concentrations of PM2.5 (from 10 μg/mL to 200 μg/mL) for different time periods, and the results showed that PM2.5 did not significantly affect the cell viability (Figure 1a). However, we observed a progressive change in the phenotype of HK-2 cells following exposure to higher levels of PM2.5. The control HK-2 cells displayed the typical feature of epithelial cells with a cobble-stone-like appearance, but those cells treated with increased concentrations of PM2.5 underwent a morphological transformation to a spindle-shaped mesenchymal phenotype. (Figure 1b). Our study then chose the PM2.5 concentration of 25 and 50 μg/mL for further experiments to know the in vitro effects of PM2.5 on HK-2 cells.

### 2.2. Cell Migration and Expression of EMT Markers of HK-2 Cells Induced by PM2.5 

**Figure 2:**
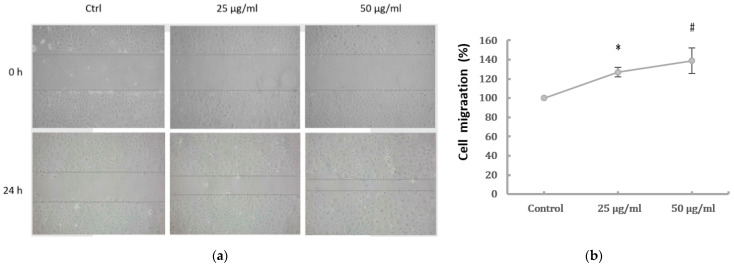

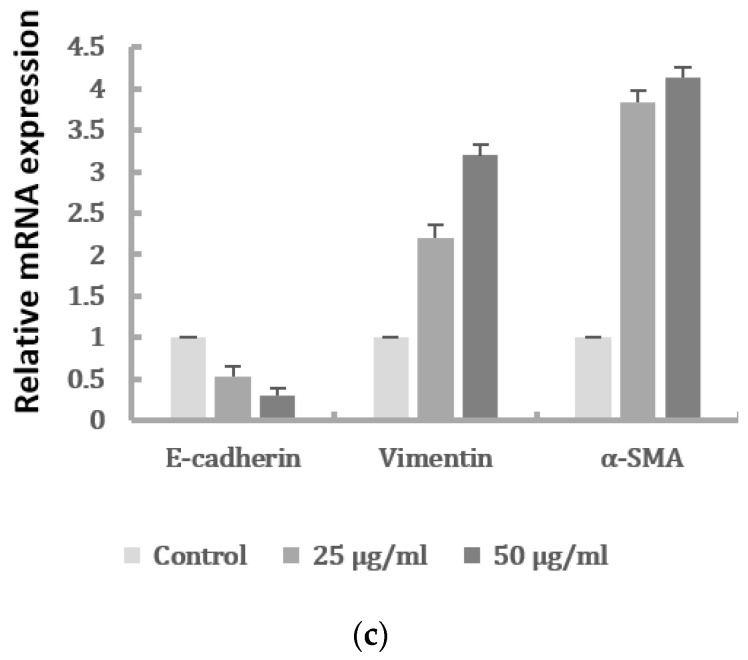
Effects of PM2.5 on cell migration and expressions of the EMT markers of HK-2 cells. (**a**) Cell migration shown by wound-healing assay (original magnification ×100). (**b**) Cell migration determined by transwell migration assay. Data represent mean ± SD. * or # means significantly different from respective controls, *p* < 0.05. (**c**) mRNAs levels of E-cadherin, vimentin, and α-SMA analyzed by qRT-PCR. Data are presented as the mean ± SD of at least three independent experiments.

PM2.5 was found to dose-dependently induce the EMT phenotype of HK-2 cells. To examine the effect of PM2.5 on HK-2 cell migration, wound healing was performed to assess whether HK-2 cells had increased migratory properties following treatment with PM2.5. The result showed that wounds treated with PM2.5 showed enhanced cell migration 24 h after wounding (Figure 2a). In addition, a transwell assay revealed that cell migration was significantly higher following PM2.5 treatment (Figure 2b). To investigate the expression of mRNAs involved in EMT, qRT-PCR was used to determine the levels of E-cadherin, vimentin, and α-SMA after PM2.5 exposure. Our data showed that PM2.5 reduced E-cadherin and increased expressions of vimentin and α-SMA (Figure 2c). These results indicate that PM2.5 could result in EMT features of HK-2 cells, characterized by the changes in phenotype and expression of EMT markers.

### 2.3. The Activation of IL-6/STAT3 Signaling Pathway in HK-2 Cells after PM2.5 Exposure

**Figure 3:**
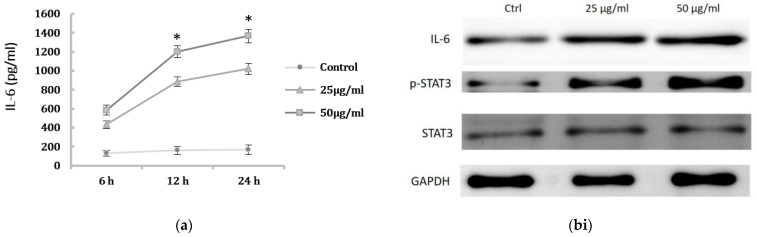

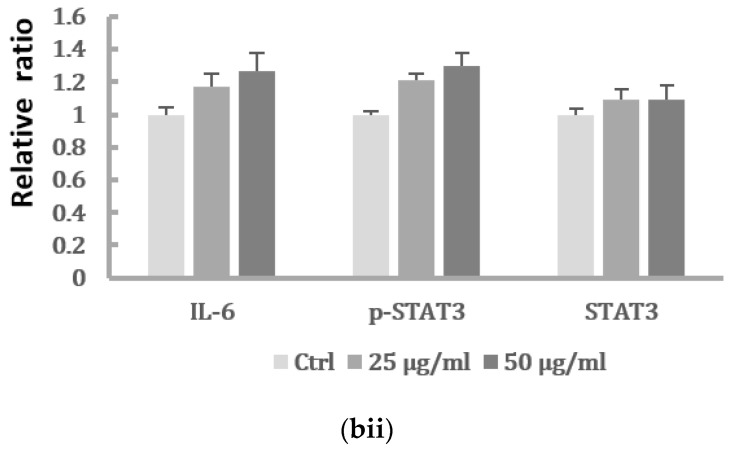
Activation of IL-6-mediated STAT3 signaling pathway induced by PM2.5. (**a**) The levels of IL-6 in supernatant of HK-2 cells induced by PM2.5 using ELISA. HK-2 cells were treated with 0, 25, 50 μg/mL PM2.5 for indicated period. Data represent mean ± SD. * means significantly different from controls, *p* < 0.05. Data are presented as the mean ± SD of at least three independent experiments. (**bi**) Western blot results showing the expression of the IL-6, p-STAT3, and STAT3 levels of HK-2 cells after PM2.5 treatment. (**bii**) The relative protein levels were quantified by densitometry and are normalized to the expression of GAPDH.

Previous study has shown pro-inflammatory cytokines may be associated with renal cell injury presenting with mesenchymal phenotype. We next determined the IL-6 levels of HK-2 cells after PM2.5 exposure. The production of IL-6 in cell culture supernatant was examined using ELISA, and the results showed the IL-6 levels were significantly increased after PM2.5 exposure (Figure 3a). We thus investigated the regulatory role of IL-6 in HK-2 cells induced by PM2.5 and assessed the expression of STAT3 and phosphorylated STAT3 protein (p-STAT3). The Western blot analysis demonstrated that IL-6 and p-STAT3 in PM2.5-treated HK-2 cells were significantly increased when compared to the control (Figure 3(bi,bii)). These results suggested that the expression of EMT in HK-2 cells induced by PM2.5 is associated with IL-6 production and STAT3 activation.

### 2.4. Suppression of IL-6 Inhibited EMT Features of HK-2 Cells Induced by PM2.5

**Figure 4:**
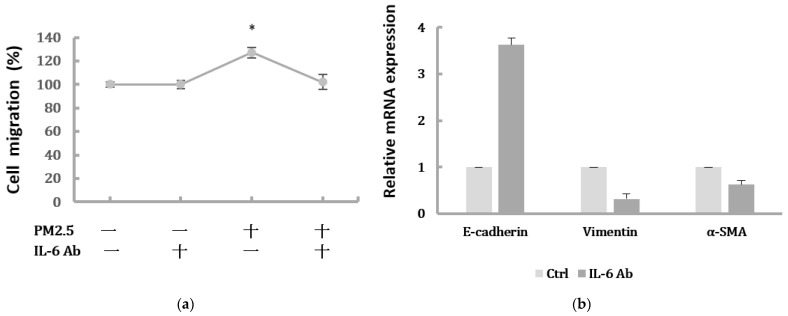
Blocking IL-6 by neutralization antibody inhibited migration and EMT markers induced by PM2.5. (**a**) Cell migration of HK-2 cells following exposure to PM2.5 then treated with IL-6-neutralizing antibody. Data are presented as the mean ± SD of at least three independent experiments. * means significantly different from controls, *p* < 0.05. (**b**) The relative expression levels of EMT-related markers after blocking IL-6 by neutralization antibody.

In order to investigate the role of IL-6 in regulating the EMT of HK-2 cells, we further determined whether inhibition of the IL-6 pathway would suppress the EMT of HK-2 cells induced by PM2.5. As shown in Figure 4a, the increased HK-2 cell migration induced by 50 μg/mL PM2.5 was suppressed by an IL-6-neutralizing antibody. In addition, blocking IL-6 by neutralization antibodies significantly inhibited the upregulation of the mesenchymal marker α-SMA and downregulation of the epithelial marker E-cadherin induced by PM2.5 (Figure 4b).

### 2.5. Inhibition of STAT3 Activation Decreased the EMT Expressions of HK-2 Cells Induced by PM2.5

**Figure 5:**
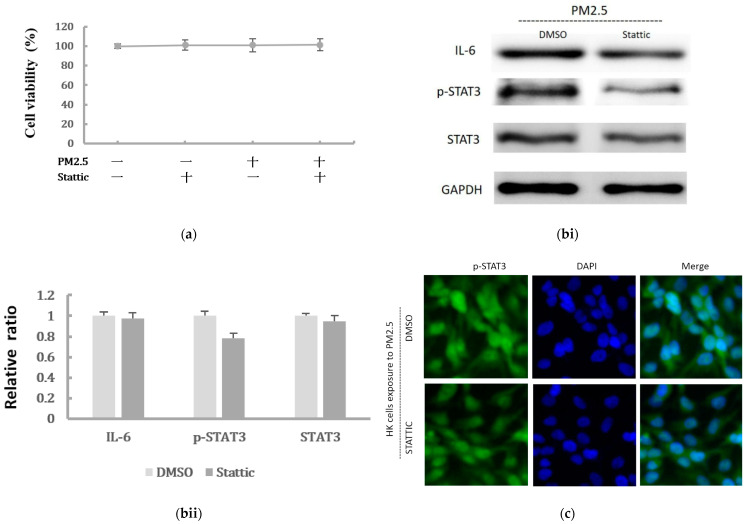

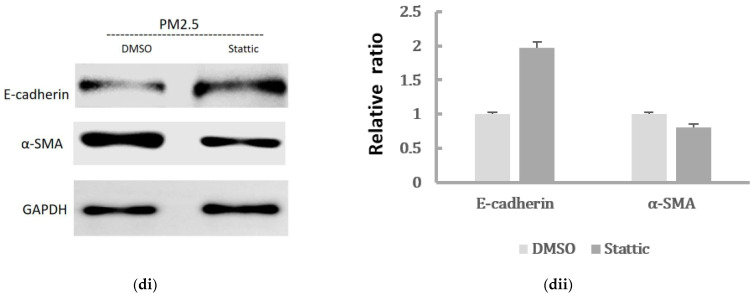
The effect of Stattic on the protein levels of IL-6/STAT3 and EMT markers induced by PM2.5. (**a**) Effect of Stattic on cell viability of HK-2 cells following exposure to PM2.5. (**bi**) IL-6 expression and STAT3 was measured by Western blotting following exposure to PM2.5 with pretreatment by Stattic. (**bii**) The relative protein levels were quantified by densitometry and are normalized to the expression of GAPDH. (**c**) Immunofluorescence evaluation of p-STAT3 in HK-2 cells following exposure to PM2.5 was reduced after pretreated by Stattic. (**di**) The EMT-related protein expression was measured by Western blotting following exposure to PM2.5 with pretreatment by Stattic. (**dii**) The relative protein levels were quantified by densitometry and are normalized to the expression of GAPDH.

To further confirm whether IL-6/STAT3 pathway was involved in the PM2.5-induced EMT change in HK-2 cells and whether STAT3 was required for the EMT-related protein expression, the STAT3 activity was inhibited by the pretreatment of a STAT3 inhibitor. Stattic was used in 5 μM to inhibit the activation of STAT3 after we confirmed it had no effect on cell viability of HK-2 cells (Figure 5a). We found that Stattic significantly inhibited the phosphorylation of STAT3, as demonstrated by Western blot (Figure 5(bi,bii)). However, it did not cause significant alternation in IL-6 production. By the immunofluorescence, the expression of p-STAT3 was markedly inhibited by Stattic in HK-2 cells exposed to PM2.5 (Figure 5c). In addition, the downregulated E-cadherin and upregulated α-SMA were remarkably reversed by Stattic, showing EMT induced by PM2.5 was decreased after STAT3 inhibition (Figure 5(di,dii)). These results suggested that PM2.5 could induce early renal tubule cell injury through the IL-6-mediated STAT3 signaling pathway.

### 2.6. Migration of Spheroids and Expression of EMT Markers in HK-2 Cells Exposure to PM2.5 by 3D Culture Models

**Figure 6:**
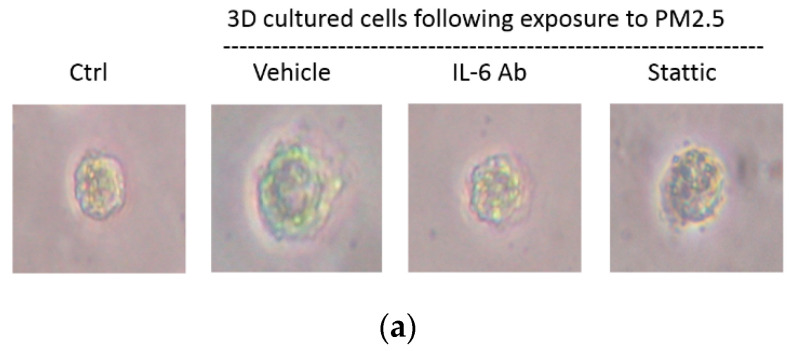

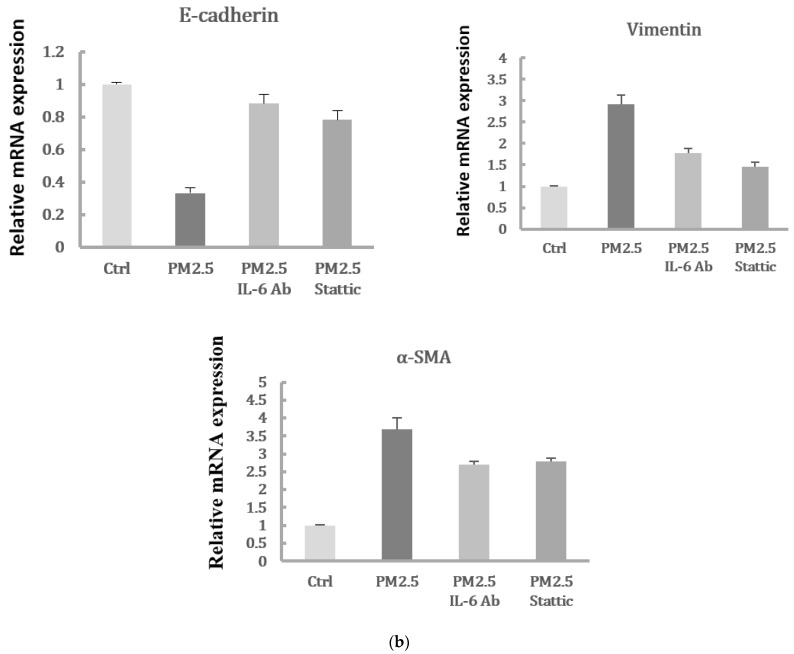
Spheroid migration and expression of EMT markers in HK-2 cells exposure to PM2.5 by 3D culture models. (**a**) Cell migratory capacity was evaluated by a spheroid invading to the Matrigel. (**b**) Expression of mRNAs levels including E-cadherin, vimentin, and α-SMA were examined by qRT-PCR.

To verify that PM2.5 could induce EMT of HK-2 cells in the physiological environment, we also used the 3D culture models to identify the migration of the cell spheroids with PM2.5 treatment. The results showed the increased level of cells moving away from spheroids to the Matrigel after PM2.5 exposure. Additionally, the treatment of IL-6-neutralizing antibodies or Stattic could lead to the suppression of cell migration and invasion caused by PM2.5 (Figure 6a). Moreover, the expression of E-cadherin was decreased, and the expressions of vimentin and α-SMA were increased after PM2.5 exposure, but the changes reduced after treatment with IL-6-neutralizing antibodies or Stattic (Figure 6b). The data using 3D cell culture models were consistent with findings employing 2D cultures; both results showed that PM2.5 could cause early EMT in tubule epithelial cells and suggested that IL-6/STAT3 signaling activation may play an important role in this process.

## 3. Discussion

Several observational studies have suggested that long-term PM2.5 exposure may affect renal function and is associated with an increased risk of kidney disease [24,25,26]. A recent report indicated the short-term, adverse effects of PM2.5 exposure on renal health [27]. In this study, we demonstrated that the toxic effects of PM2.5 could lead to notable EMT features in HK-2 cells by the regulation of IL-6/STAT3 pathway. PM2.5 induced the mesenchymal phenotype of renal tubular epithelial cells, as observed by an enhanced migratory capacity, and a change in EMT-related mRNA expression levels, including increased vimentin and α-SMA expression and decreased E-cadherin expression.

EMT is a process wherein epithelial cells express spindle-like cell morphology. It is associated with loss of cell adhesion and an increase in cell migration. Some studies have shown that PM2.5 correlates with EMT initiation and accounts for alterations in pulmonary epithelial cell morphology and fibrosis progression [28,29]. In renal pathological conditions, EMT of renal tubular epithelial cells may be an early event in renal fibrosis [30]. Renal tubule cells undergoing EMT could express the downregulation of epithelial proteins, such as E-cadherin and upregulation of mesenchymal markers, including vimentin and α-SMA [31]. Our results showed that HK-2 cells express EMT features following PM2.5 exposure, and this change was associated with increased IL-6 levels and elevated STAT3 activation.

When we inhibited IL-6 with neutralizing antibodies, the migration and expression levels of EMT-related mRNA in HK-2 cells were significantly attenuated. Furthermore, we found that the administration of Stattic, a STAT3 inhibitor, could inhibit STAT3 phosphorylation, whereas there were no significant alterations in IL-6 levels. E-cadherin and α-SMA were markedly reversed by Stattic, showing an ameliorative effect on PM2.5-induced EMT after STAT3 inhibition. These findings suggest that PM2.5 can upregulate IL-6-mediated STAT3 expression. Also, the activation of the IL-6/STAT3 pathway was associated with the EMT process of HK-2 cells. Previous studies have shown that IL-6 production is involved in the airway inflammatory response and PM2.5-induced respiratory diseases [32,33]. Additionally, the roles of IL-6 were reported in several kidney diseases and targeting IL-6 could protect against renal fibrosis by suppressing STAT3 activation [34,35]. Some studies have found that STAT3 signaling can control the EMT of renal tubular epithelial cells during renal injury, and dysregulated STAT3 may lead to renal disease, including inflammation and fibrosis [36,37].

By the application of 3D cell models to better mimic in vivo cells behaviors, we found that PM2.5 enhanced the migratory behavior of spheroids cultured in the Matrigel matrix. Administration of IL-6-neutralizing antibodies or Stattic significantly attenuated the migratory capacity of spheroids invading to the Matrigel. Additionally, the expression of EMT markers revealed by qRT-PCR further validated the migratory phenotype of the 3D spheroids. Overall, the models using monolayer and 3D cell cultures both showed that exposure to PM2.5 resulted in early EMT in tubule epithelial cells through the activation of IL-6/STAT3 signaling pathway. The limitations of the present study do exist due to the in vitro situation. However, our results may provide useful support data for further in vivo study evaluation.

Taken together, our study revealed that PM2.5 could induce HK-2 cells to express early EMT phenotype through activation of IL-6/STAT3 pathway, suggesting that targeting IL-6/ STAT3 pathway may have the potential to attenuate the harmful effects of PM2.5. Our findings indicated the early events and possible mechanism of PM2.5 on tubule epithelial cells. Further study to determine if long-term exposure to PM2.5 could contribute to tubulointerstitial fibrosis and renal dysfunction is warranted.

## 4. Materials and Methods

### 4.1. Cell Culture and Materials

Human proximal tubular epithelial cell line (HK-2 cells) was cultured in DMEM/F12 medium supplemented with 10% FBS in a humidified atmosphere of 5% CO_2_ at 37 °C. Cells were serum starved overnight before treatment with PM2.5 for the indicated times. The PM2.5 (diesel particulate matter) was purchased from Sigma-Aldrich Chemical Co. (Austin, TX, USA). A 50 mg/mL stock solution of PM2.5 was prepared in DMSO (Invitrogen-Gibco, Carlsbad, CA, USA) and diluted to the required concentrations in culture medium before use. IL-6-neutralizing antibodies were purchased from R&D Systems (Minneapolis, MN, USA). Stattic was purchased from Selleck Chemicals (Houston, TX, USA).

### 4.2. Quantification of IL-6 Levels by Enzyme-Linked Immunosorbent Assay (ELISA)

The supernatants of cell lines were quantitated using ELISA assay for IL-6 levels measurement (R&D Systems, Minneapolis, MN, USA) according to manufacturer instructions. Triplicated samples were estimated, and the averaged values were normalized to total cell counts in the analysis.

### 4.3. Cell Viability Assay

The cell viability of human HK-2 cells was examined by MTT assay. HK-2 cells with 10,000 per well were seeded in 96-well plates and allowed to grow for 24 h. After exposure to different concentrations of PM2.5 (0, 10, 25, 50, 100, and 200 μg/mL) for 24 h, 48 h, and 72 h, the culture medium was removed, and the cells were washed twice in PBS and incubated in 50 μL MTT reagent (0.5 mg/mL). After incubation for 4 h at 37 °C, 100 μL of DMSO was added to each well and the absorbance was measured by a microplate reader at a wavelength of 570 nm.

### 4.4. Wound Healing and Transwell Migration Assays

Wound healing assay: the cell monolayer was scratched with a micropipette and photographed at the beginning of the assay at 0 h and at 24 h with or without PM2.5. Transwell migration assay: performed using the polycarbonate filter membrane (24-well insert, pore size 8 μm, Corning Costar, 07-200-150). Cells were seeded to a density of 50,000 cells per well and incubated for 24 h, then exposed to PM2.5 in respective conditions. The non-migratory cells were removed, and the cells on the lower surface of the membrane were fixed with methanol for 10 min and stained with 0.5% crystal violet for 30 min. The number of stained cells in five randomly selected fields was counted.

### 4.5. RNA Isolation and Quantitative Real-Time Polymerase Chain Reaction (qRT-PCR)

The total RNA was isolated from cultured cells using a Trizol reagent (Invitrogen) and reverse transcribed into cDNA using the PrimeScript RT reagent kit according to the manufacturer’s instructions. mRNA levels of target genes were quantified and normalized to GAPDH using SYBR Green Master Mix with ABI PRISM 7900 system (Applied Biosystems, Foster City, CA, USA).

The primer sequences used for the qRT-PCR were as follows:

GAPDH, Forward: 5’-ACGGGAAGCTCACTGGCATGG-3’; Reverse: 5’-GGTCCACCACCCTGTTGCTGTA-3’; E-Cadherin, Forward: 5’-TGCCCAGAAAATGAAAAAGG-3’; Reverse: 5′-GTGTATGTGGCAATGCGTTC-3′; Vimentin, Forward: 5’-GAGAACTTTGCCGTTGAAGC-3’; Reverse: 5’-GCTTCCTGTAGGTGGCAATC-3’; α-SMA, Forward: 5’-CTATGCCTCTGGACGCACAACT-3’; Rverse: 5’-CAGATCCAGACGCATGATGGCA-3’.

### 4.6. Western Blot Analysis

Protein extracted from cells was separated on 10% SDS-polyacrylamide electrophoresis gels and transferred to a polyvinylidene difluoride membrane and immunoblotted with specific antibodies. The primary antibodies included mouse monoclonal STAT3, rabbit monoclonal phosphorylated (p-STAT3), α-smooth muscle actin (α-SMA; Sigma, St. Louis, MO, USA), monoclonal rat IL-6, rabbit GAPDH, and mouse monoclonal vimentin (Abcam, Cambridge, MA, USA). Anti-GAPDH antibody was used as a sample-loading control. Immunoreactive protein bands were detected with the ECL detection system.

### 4.7. Immunofluorescence Staining

Cells were cultured on sterile glass coverslips in 6-well plates and fixed in 4% paraformaldehyde. After washing with PBS, the cells were permeabilized using 0.1% Triton X-100 solution for 15 min and blocked with 5% BSA for 30 min. The slides were incubated overnight at 4 °C with the primary antibody p-STAT3, followed by incubation with the fluorescent-labeled secondary antibody at room temperature for 1 h. Expression of p-STAT3 was detected and stained using the secondary Alexa 488 fluorescent-labelled antibody. The nuclei were detected by DAPI staining. The levels of protein expression were quantified by densitometry using Image J software and normalized to GAPDH.

### 4.8. Cell Migration Assessed by 3D Cell Spheroids Culture

HK-2 cells were plated into 96-well ultralow attachment plates at the density of 5 × 10^3^ cells per well. The plates were centrifuged and allowed cells to grow and form spheroids in DMEM/F12. Spheroid formation was monitored at 8 h intervals over a 3 day period. After treating the cells forming spheroids with PM2.5, the spheroids were embedded in 10% (*v*/*v*) Matrigel (Sigma-Aldrich) at 37 °C for 30 min, allowing the Matrigel to polymerize. Spheroids with PM2.5 exposure were continuously cultured or subsequently added with IL-6-neutralizing antibody or Stattic in DMEM/F12 supplemented with 10% FBS and 1% PSA for 7 additional days.

### 4.9. Statistical Analysis

The data represent the mean ± standard deviation (SD) of a minimum of three independent experiments, each performed in triplicate, and are presented relative to the control. Comparisons between the two groups were made using the Student’s *t*-test. The differences among 3 groups were analyzed by one- or two-way ANOVA. *p*-values less than 0.05 were considered statistically significant. Statistical analyses were performed with SPSS for Windows version 19.0.5.

## 5. Conclusions

PM2.5 induces early tubular epithelial injury by upregulating IL-6/STAT3 pathway and promotes EMT of renal tubular cells, suggesting that exposure to PM2.5 may increase the risk of developing kidney disease.

## Data Availability

Data will be available if requested.
